# Roles of Piezo channels in dentofacial deformity: Onset, orthodontic treatment, and associated diseases

**DOI:** 10.1016/j.gendis.2026.102123

**Published:** 2026-03-05

**Authors:** Jinhan Nie, Yi Zhang, Jun Ma, Min Hu, Huichuan Qi

**Affiliations:** a Department of Orthodontics, Hospital of Stomatology, Jilin University, Changchun, Jilin 130021, China; b Department of Oral Anatomy and Physiology, Hospital of Stomatology, Jilin University, Changchun, Jilin 130021, China; c Key Laboratory of Pathobiology, Ministry of Education, Jilin University, Changchun, Jilin 130021, China

**Keywords:** Dentofacial deformity, Mechanical stimulation, OTM, Periodontitis, Piezo, TMJOA

## Abstract

The onset and treatment of dentofacial deformities are closely associated with mechanical stimuli. As mechanosensitive ion channels, Piezo channels play a critical role in the mechanotransduction processes of various tissues and organs, including the craniofacial skeleton and periodontium. This review comprehensively examines the significant role of Piezo channels in dentofacial deformities, addressing their pathogenesis, orthodontic treatment, and the progression of related diseases. It synthesizes evidence that highlights the clinical importance of targeting Piezo channels, which can simultaneously facilitate orthodontic tooth movement, manage treatment complications, and control pain. This work enhances our understanding of the etiology of dentofacial deformities and serves as a valuable reference for clinical orthodontic practice.

## Introduction

Dentofacial deformity, among the most prevalent conditions in the oral and craniofacial systems, impacts the facial aesthetics and oral function of more than half of the global population.^1^ Craniofacial development is an intricate process of bone and cartilage growth, finely regulated by physiological stimuli encompassing biology, chemistry, and mechanics.^2^ Hence, the dysregulation of mechanical stimuli emerges as a risk factor for the onset of dentofacial deformities. As the primary treatment for mild or moderate dentofacial deformities, orthodontic treatment is a process of bone remodeling in response to mechanical stimulation.^3^ Orthodontic force regulates cell metabolism and neurovascular function, impacting local bone resorption and deposition, thereby regulating skeletal reshaping and tooth movement.^4^^,^^5^ Furthermore, various parts of the craniofacial complex are closely interrelated. Dentofacial deformities have been demonstrated to be associated with temporomandibular joint osteoarthritis (TMJOA) and periodontitis^6^^,^^7^ due to mechanical overloading on the temporomandibular joint or periodontal tissue resulting from abnormal occlusion.^8^^,^^9^ Therefore, mechanical stimulation plays a significant role in the onset and treatment of dentofacial deformities, along with the progression of associated diseases. Nonetheless, the mechanisms of mechanotransduction remain unclear.

**Table 1:** Piezo-specific knockout mouse models.

Cell	Whole body phenotype	Craniofacial skeletal phenotype
Neural crest cells^27^		Defects in jawbone development
Osteoblastic lineage cells/*OcnCre*^29^	Reduced bone strength and bone mass	Incomplete closure of the cranial suture
Osteoblastic lineage cells/*OsxCre*^31^	Reduced trabecular and cortical bone mass	
Osteoblastic lineage cells/*Dmp1Cre*^30^	Reduced trabecular bone mass and bone stiffness; spontaneous fractures	
Osteoblastic lineage cells/*Dmp1Cre*^44^	Reduced trabecular and cortical bone mass	
Osteoblastic lineage cells/*Runx2Cre*^32^	Reduced trabecular mass	Reduced autonomous osteogenesis in cranial progenitor osteoblasts
Chondrocytes/*Col2a1Cre*^32^	Reduced trabecular bone volume	

Recently, the effect of mechanosensitive ion channels in mechanotransduction has increasingly come to light.^10^ First identified as a tactile receptor, Piezo channels were revealed to be conserved mechanically activated ion channels across evolution.^11^^,^^12^ Studies have demonstrated that Piezo channels can be activated by various forms of mechanical force and are involved in numerous physiological activities.^11^ Furthermore, in vertebrates, the two members of the Piezo family, Piezo1 and Piezo2, function in different tissues and organs.^10^ Piezo1 is widely expressed in bone, cartilage, the immune system, the cardiovascular system,^11^^,^^13^^,^^14^ etc., whereas Piezo2 is primarily expressed in sensory neurons.^15^ More recently, Piezo proteins were also detected in the craniofacial skeleton and periodontal tissue,^16^^,^^17^ indicating a possible role of Piezo proteins in converting mechanical stimuli into intercellular response in the craniofacial complex.

**Figure 1:**
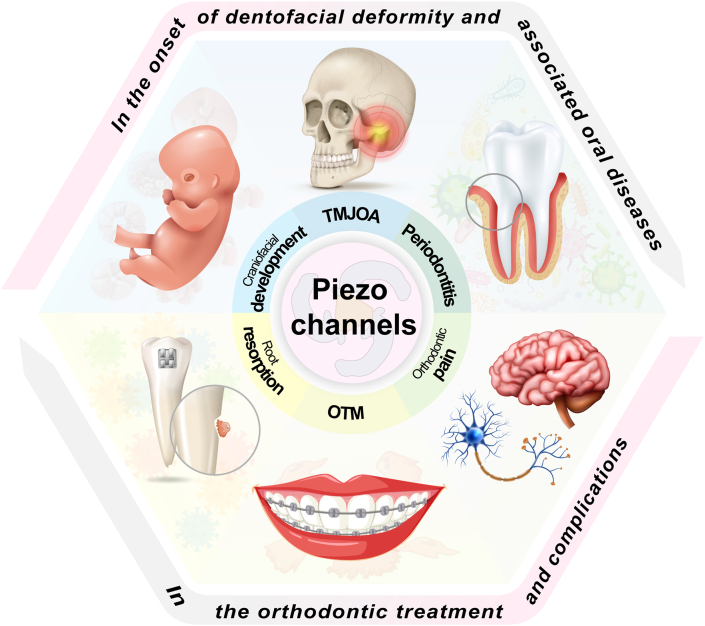
The role of Piezo channels in dentofacial deformity. Piezo channels regulate craniofacial development, and Piezo protein deficiency is associated with dentofacial deformities. Piezo channels also mediate the development of associated diseases, such as temporomandibular joint osteoarthritis (TMJOA) and periodontitis, in the state of dentofacial deformity. In addition, Piezo channels are key mediators of orthodontic tooth movement (OTM) and are involved in the progression of orthodontic complications.

This review explores the significance of Piezo channels in dentofacial deformities, focusing on their involvement in disease onset, orthodontic treatment, and the progression of related conditions such as TMJOA and periodontitis (Fig. 1). This study emphasizes the mechanical mechanisms that are common to the pathological, treatment, and complication processes associated with dentofacial deformities. Building on this integrated understanding, it underscores the pivotal potential of Piezo channels in advancing clinical orthodontic practice.

## The structure and activation of Piezo channels

Piezo proteins are highly conserved, large proteins with an estimated 24 to 36 transmembrane domains and containing 2100 to 4700 amino acids.^18^ Regarding the amino acid sequence, Piezo1 and Piezo2 display 42% sequence homology.^18^ Whereas structurally, they both demonstrate homotrimeric structures, albeit with notable differences.^18^

In 2015, a three-blade, propeller-shaped architecture of mouse Piezo1 protein was first observed using medium-resolution cryo-electron microscopy.^19^ Piezo1 comprises a peripheral mechanotransduction module and a central ion conduction pore module, forming a lever-like mechanotransduction mechanism.^19^^,^^20^ Within the peripheral mechanotransduction module, flexible blades act as potential force sensors, while a beam functions as a lever-like device to couple the distal blades to the central pore.^19^ The central ion conduction pore module can mediate inward cation flow in response to altered lateral cell membrane tension,^21^^,^^22^ translating various mechanical stimuli into physiological cellular activity.^23^

In contrast to Piezo1, charged amino acids in Piezo2 are present at the interface between the beam and the C-terminal domain (CTD) of the central ion conduction pore module.^24^ The neutralization of these specific residues leads to an increased threshold for mechanical stimulation in Piezo2.^24^ Additionally, an extra constriction between the inner helices of Piezo2 results in a smaller central pore compared with Piezo1.^24^^,^^25^ These structural disparities between Piezo1 and Piezo2 suggest that the transmembrane site may serve as a gate to regulate channel mechanosensitivity.^24^

## Effects of Piezo channels in the onset of dentofacial deformity

Abnormal craniofacial development caused by specific factors or hereditary influences significantly contributes to the onset of dentofacial deformities.^26^ A recent study has demonstrated the jaw deformities resulting from Piezo channels deletion.^27^ Specifically, Piezo1 regulates the migration of neural crest stem cells.^28^ Inhibiting Piezo1 led to the overactivation of Rac1, abnormal invasion of neural crest stem cells,^28^ and ultimately induced jaw deformities in mice.^27^ The simultaneous deletion of Piezo1 and Piezo2 led to more severe jaw defects than those observed in the single knockout model, indicating a critical yet partially redundant role for Piezo1 and Piezo2.^27^

In addition to abnormalities in neural crest stem cell migration, Piezo1 and Piezo2 double knockout mice exhibited excessive osteoblast death in the maxillary and mandibular jaw.^27^ Piezo channels within the osteoblastic lineage cells contribute to bone development throughout the body, including the craniofacial region (Table 1). Mice with conditional knockout of Piezo1 in osteoblastic lineage, using *OsxCre*, *Dmp1Cre*, and *OcnCre*, exhibited similar reductions in trabecular bone mass and femoral cortical thickness.^29^, ^30^, ^31^ Especially, Piezo1^*OcnCre*^ mice displayed incomplete closure of the cranial suture,^29^ and reduced autonomous osteogenesis was observed in cranial progenitor osteoblasts isolated from Piezo1^*Runx2Cre*^ mice.^32^ While craniofacial bone development is primarily governed by intramembranous osteogenesis, endochondral osteogenesis also plays a critical role.^33^ Deletion of Piezo channels in chondrocytes impacts skeletal development, as evidenced by the skeletal developmental issues and increased fracture risk observed in Piezo1^*Col2a1Cre*^ mice, but no specific deformities in craniofacial development were reported.^32^

In summary, Piezo1 plays a significant role in craniofacial development by regulating neural crest stem cell migration, osteoblastic lineage activity, and endochondral ossification in synergy with Piezo2. Dysfunctional Piezo channels would lead to dentofacial deformities.

## Effects of Piezo channels in orthodontic treatment

### Regulation of osteogenic and osteoclastic cells during OTM

**Figure 2:**
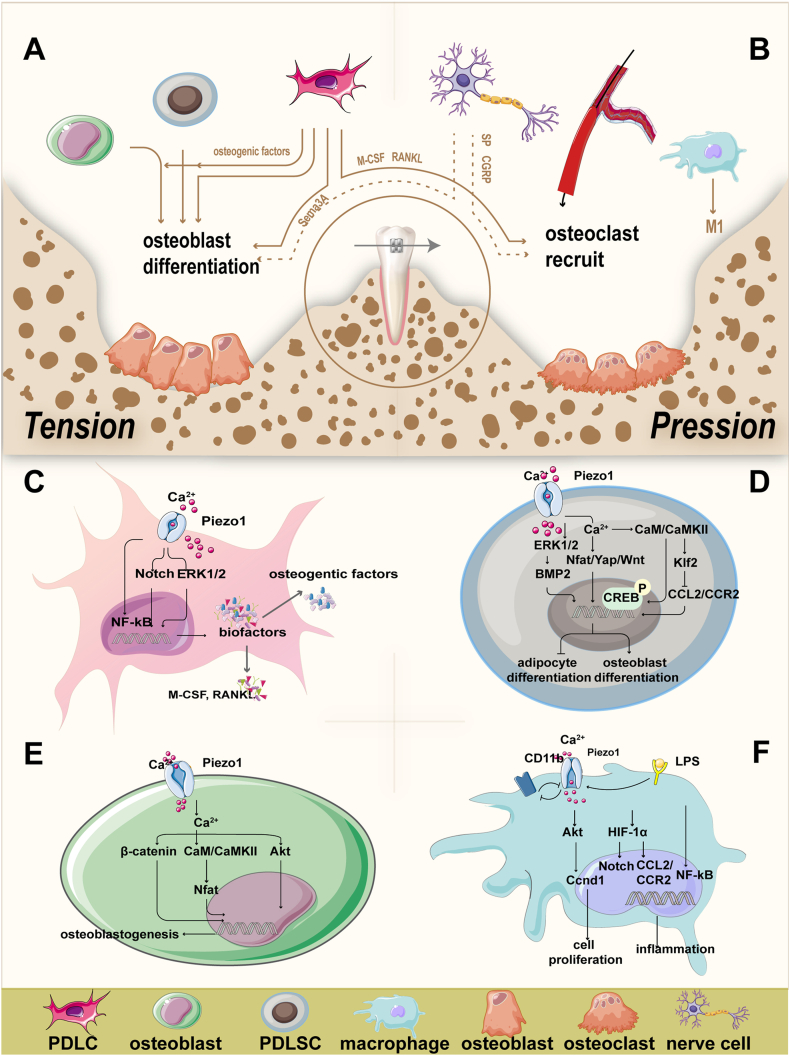
The role of Piezo channels in orthodontic tooth movement. **(A)** Piezo-mediated cellular activity on the tension side is predominantly characterized by osteoblastogenesis. Under Piezo1-mediated tension, periodontal ligament cells (PDLCs), periodontal ligament stem cells (PDLSCs), and pre-osteoblasts all undergo differentiation into osteoblasts. Moreover, PDLCs concurrently secrete osteogenic factors that facilitate osteogenic differentiation. Neuronal cells expressing Piezo1/2 may also play a role in promoting osteogenic differentiation by secreting Sema3A. **(B)** Piezo-mediated cellular activity on the pressure side is predominantly characterized by osteoclastogenesis. Under Piezo1-mediated pression, PDLCs secrete osteoclastogenic factors (M-CSF, RANKL), which recruit osteoclasts from the peripheral blood. Additionally, macrophages polarize towards M1, and neuronal cells might further exacerbate local inflammation and expedite osteoclastogenesis by secreting SP and CGRP. **(C)** The role of Piezo1 and downstream pathways in PDLCs. In response to mechanical force stimulation, the opening of Piezo1 channels results in the inward flow of Ca^2+^, which activates downstream ERK1/2 or Notch channels, leading to the secretion of osteogenic factors. Moreover, this process causes NF-κB nuclear translocation and the release of osteoclastogenic factors (M-CSF, RANKL). **(D)** The role of Piezo1 and downstream pathways in PDLSCs. The opening of Piezo1 channels activates downstream ERK1/2, Wnt, and CaM/CaMKII pathways, contributing to osteoblast differentiation, inhibiting adipogenic differentiation. **(E)** The role of Piezo1 and downstream pathways in osteoblasts. The opening of Piezo1 channels activates downstream Wnt/β-catenin, CaM/CaMKII, and Akt pathways, contributing to osteoblastogenesis. **(F)** The role of Piezo1 and downstream pathways in macrophages. The crosstalk between Piezo1 and CD11b triggers downstream Akt pathways, leading to cell proliferation. Furthermore, the activation of Piezo1 promotes HIF-1α/Notch, CCL2/CCR2, and NF-κB pathways and mediates cell polarization towards the M1 phenotype. M-CSF, myeloid colony-stimulating factor; RANKL, receptor activator of nuclear factor-κB ligand; Sema3A, Semaphorin3A; SP, substance P; CGRP, calcitonin gene-related peptide; CaM, calmodulin; CaMKII, calmodulin-dependent protein kinase II; NFAT1, nuclear factor of activated T cells 1; HIF-1α, hypoxia-inducible factor-1α; CCL2, C–C motif chemokine 2; CCR2, C–C motif chemokine receptor 2; ERK, extracellular signal–regulated kinase; NF-κB, nuclear factor κB; BMP, bone morphogenetic protein; YAP, yes-associated protein; PI3K, phosphatidylinositol-3 kinase; Akt, protein kinase B; Klf2, kruppel-like factor 2.

Orthodontic tooth movement (OTM) is essentially a process of bone remodeling under mechanical action.^34^ Applying orthodontic forces induces the division of periodontal tissue into tension and pressure sides, leading to sequential osteoblastogenesis and osteoclastogenesis in distinct mechanical environments.^34^ This process effectively initiates the restructuring of alveolar bone, ultimately resulting in tooth movement.^34^ Piezo channels are particularly important in this process. Strong immunolocalization of both Piezo1 and Piezo2 was observed in the periodontal ligament of both humans and mice.^16^ Piezo1 has demonstrated significant up-regulation under orthodontic forces in a rat OTM model.^35^^,^^36^ Inhibiting Piezo1 or Piezo2 resulted in a significantly reduced rate of tooth movement.^35^, ^36^, ^37^ The high involvement of Piezo channels in the OTM is attributed to their expression and function across various cell types within periodontal tissues^35^^,^^36^ (Fig. 2).

### Piezo mediates PDLC mechanical response

Within the periodontal ligament, periodontal ligament cells (PDLCs) are the most prevalent mechanosensitive cells.^38^ PDLCs receive orthodontic forces and produce cytokines to regulate the proliferation and differentiation of pluripotent cells.^38^ Acting as “sentinel cells”, PDLCs convert mechanical stimuli into biochemical signals, thereby modulating osteogenic and osteoclastic activity. Notably, PDLCs also possess the capacity to differentiate into osteoblast-lineage cells.^39^ Evidence indicates that these functions are partly mediated by Piezo1, whose expression in PDLCs is up-regulated under mechanical stress.^40^ This up-regulation is associated with enhanced osteogenic and osteoclastic induction. Specifically, the expression of osteoclast-inducing factors, including macrophage colony-stimulating factor (M-CSF), receptor activator of nuclear factor-κB ligand (RANKL), and prostaglandin E2 (PGE_2_), as well as osteogenic transcription factors, correlates positively and significantly with Piezo1 levels.^40^^,^^41^ These findings confirm that orthodontic forces activate the Piezo1 channel in PDLCs, triggering the release of osteogenic and osteoclastic mediators and thereby regulating bone formation and resorption.

### Piezo promotes osteogenic differentiation

In addition to the plentiful PDLCs, osteoblasts in the periodontal tissue are directly involved in bone remodeling. Piezo1 is extensively expressed in various types of osteoblastic lineage cells, covering osteoblast progenitors, osteoblasts, and osteocytes, and plays a crucial role in mechanotransduction.^11^ The RNA-sequencing analysis in osteocytes showed a significantly higher expression of Piezo1 in the fluid flow groups compared with the static groups.^30^ Similarly, in the pre-osteoblasts, mechanical stimulation enhances the release of osteogenesis-related factors through up-regulating the expression of Piezo1.^42^ Inhibiting Piezo1 can partially inhibit the enhancement of osteoblastic capacity.^29^^,^^42^ It is noteworthy that mechanical stimulation not only boosts local osteoblasts through Piezo1 activation but also concurrently inhibits osteoclast activity in the region. For instance, in osteocytes, mechanical stimulation activates Piezo1-mediated osteogenic responses alongside suppressed sclerostin (Sost) expression.^43^ The response that could be reversed by treating with the Piezo1 inhibitor GsMTx4.^43^ Similarly, Piezo1^*Prrx1Cre*^ mice exhibit impaired osteogenic capacity concurrent with markedly enhanced osteoclastic activity.^44^ Therefore, Piezo1 channels in osteoblast cell lines regulate osteogenic activity and modulate local osteoclasts through the intricate crosstalk between osteoblasts and osteoclasts.^44^

### Piezo recruits osteoclasts

Osteoclastogenesis plays a crucial role in the OTM process, marking the commencement of bone remodeling and deciding the speed of tooth movement.^45^ Given its significance, there is considerable interest in examining whether Piezo1 directly affects osteoclasts. Research has shown that in pre-osteoclastic cells, the mechanical response was less pronounced, and Piezo1 expression was markedly lower compared to pre-osteoblasts.^29^ Additionally, the conditional knockout of Piezo1 in osteoclastic lineage cells did not lead to abnormal bone metabolism, as observed in both Piezo1^*CtskCre*^ and Piezo1^*lyz2Cre*^ mice.^32^^,^^44^ Based on current evidence, it can be concluded that the expression and function of Piezo1 in the osteoclastic lineage cells are less abundant than in the osteoblastic lineage cells. Studies have demonstrated that osteoclasts located in periodontal tissue are primarily recruited from the peripheral blood,^46^ governed by two main factors: M-CSF and RANKL.^47^^,^^48^ Therefore, the alterations in the number and function of osteoclasts during OTM are more likely attributable to the release of soluble biomolecules by mechanosensitive cells, rather than representing their direct response to mechanical forces. In summary, although Piezo1 does not directly regulate osteoclast function, it initiates the release of regulatory factors that modulate osteoclast activation and activity indirectly through paracrine signaling. This positions Piezo1 as an upstream regulator in the control of osteoclastic processes.

### Piezo determines the differentiation direction of PDLSCs

As a specialized cellular component of the periodontal ligament, periodontal ligament stem cells (PDLSCs) are also involved in the OTM process.^49^ Like mesenchymal stem cells from other sites, PDLSCs possess multilineage differentiation potential.^50^ Mechanical stimulation or Yoda1-induced activation of Piezo1 promotes the bidirectional differentiation of human PDLSCs toward osteogenic and cementogenic lineages.^51^ Upon Piezo1 channel activation, calcium influx induces CAMP-responsive element-binding protein (CREB) phosphorylation,^51^ leading to up-regulated expression of osteogenic and cementogenic proteins.^51^, ^52^, ^53^ Furthermore, Piezo1 enhances the mineralization capacity of PDLSCs.^51^ Conversely, genetic knockout or pharmacological inhibition of Piezo1 significantly impairs their osteogenic and cementogenic differentiation potential.^51^^,^^54^ Therefore, Piezo1 emerges as a pivotal molecule influencing the fate of PDLSCs during OTM.

Mechanical forces activate the Piezo1 channel in periodontal cells, triggering a cascade of downstream signaling pathways that regulate cell behavior and intercellular communication. This regulation maintains the dynamic balance between osteogenic and osteoclastic activity during OTM. The nuclear factor kappa B (NF-κB) pathway is activated by the Piezo1 pathway, promoting the release of osteoclastogenic factors and recruiting osteoclasts.^40^ Following Piezo1 activation, Ca^2+^ acts as the fastest responding second messenger. The influx of Ca^2+^ stimulates calmodulin (CaM) and its downstream effector, Ca^2+^/calmodulin-dependent protein kinase II (CaMKII).^55^^,^^56^ In mesenchymal stem cells, CaMKII activates kruppel-like factor 2 (Klf2), which subsequently inhibits the C–C motif chemokine 2 (CCL2)/NF-κB/lipocalin-2 (Lcn2) inflammatory autocrine pathway, promoting the differentiation of cells towards osteogenesis and inhibiting adipogenesis.^57^ It also phosphorylates CREB, enhancing the expression of osteogenesis-related genes.^51^^,^^58^ In osteocytes, CaMKII improves the lacunar–canalicular network and suppresses sclerostin synthesis.^55^^,^^56^ The Ca^2+^/CaM complex further activates nuclear factor of activated T cells 1 (NFAT1), which synergistically promotes osteogenic differentiation and coupled angiogenesis.^59^ Additionally, Piezo1 activation modulates hemichannels composed of connexins such as connexin43 (Cx43) and pannexin 1 (Panx1), enabling ATP release into the extracellular space.^60^ Extracellular ATP stimulates P2X receptors, prolonging and amplifying intracellular Ca^2+^ signaling.^60^ This feedback loop supports sustained bone anabolism. At the transcriptional level, mechanical forces induce nuclear translocation of yes-associated protein (YAP) and transcriptional coactivator with PDZ-binding motif (TAZ) via Piezo1.^61^, ^62^, ^63^ These coactivators adjust osteogenic gene expression programs. Piezo1 also activates the canonical Wnt/β-catenin pathway, directly initiating osteogenic transcription.^64^ Furthermore, Piezo1 triggers phosphorylation cascades through phosphatidylinositol-3 kinase (PI3K)/protein kinase B (Akt)^60^ and extracellular signal-regulated kinase (ERK)^41^ pathways. These cascades promote cell proliferation, differentiation, and survival. In summary, Piezo1 and its multilevel downstream network provide spatiotemporally precise regulation of alveolar bone remodeling during OTM.

### Mediating immune cell regulation during OTM

OTM is characterized as an aseptic inflammatory process.^49^ Immune cells, including macrophages and T cells, when exposed to orthodontic forces, actively participate in alveolar bone remodeling.^65^

### Piezo regulates macrophage polarization

Under mechanical regulation, macrophages accumulate within the periodontal tissue, and their polarization state is concurrently modulated.^66^ Orthodontic forces initially contribute to the accumulation of M1-like macrophages.^65^ Analogous to osteoclast function, the inflammatory polarization of macrophages accelerates tooth movement.^67^ Following orthodontic release, a reversal occurs in the M1/M2 ratio, and M2 macrophages assume a pivotal role in tissue repair.^68^ The Piezo1 channel serves as a primary contributor to macrophage responses to orthodontic force.^69^ In response to local inflammatory factors or orthodontic forces, macrophages exhibit augmented Piezo1 expression, up-regulation of intracellular calcium ion concentration, increased proliferative capacity, and a more pronounced M1 phenotype.^70^, ^71^, ^72^ Mechanical strain-induced activation of Piezo1 in macrophages increases the expression of inflammatory cytokines and osteoprotegerin (OPG),^73^ and triggers activation of the NOD-like receptor thermal protein domain–associated protein 3 (NLRP3) inflammasome.^74^ In contrast, pharmacological or genetic blockade of Piezo1 markedly reduces macrophage responsiveness to mechanical stimulation.^73^

### Piezo affects T cell function

T cells play a pivotal role in OTM. In immunodeficient mice with impaired T-cell function, the number of tartrate-resistant acid phosphatase (TRAP)-positive osteoclasts in the periodontal tissue is significantly reduced, accompanied by a shorter distance of tooth movement compared with wild-type controls.^75^ Intravenous transfer of T cells restores the osteoclast population in the OTM region of immunodeficient mice.^75^ Within the periodontal ligament, γδ T cells outnumber αβ T cells.^76^ Conditional deletion of γδ T cells *in vivo* suppresses interleukin-17 (IL-17) production, leading to diminished osteoclast formation and impaired OTM.^76^ Under mechanical loading, polarization of Th17 cells is enhanced, thereby amplifying local inflammatory responses in the periodontium.^77^ Interestingly, mechanical strain-induced activation of Piezo1 strengthens mesenchymal stem cell–T cell communication via a glycolytic pathway, promoting Th17 polarization in a macrophage migration inhibitory factor (MIF)-dependent manner.^78^ In contrast, regulatory T (Treg) cells exhibit a negative correlation with receptor activator of RANKL expression during OTM.^79^ The Th17/Treg ratio modulates osteoclast activity and governs OTM by altering the RANKL/OPG balance.^77^^,^^79^ Piezo1 selectively suppresses Treg cells,^80^ and genetic deletion of Piezo1 in T cells expands the Treg cells population, consequently attenuating local inflammatory responses.^80^

Although the mechanisms by which Piezo1 regulates T cell differentiation under mechanical stimulation remain to be fully elucidated, current evidence suggests that Piezo1 activation suppresses Treg polarization by inhibiting transforming growth factor-β (TGF-β) signaling.^80^ In macrophages, mechanical strain-induced Piezo1 activation triggers Ca^2+^ influx, which activates calpain and markedly up-regulates hypoxia-inducible factor-1α (HIF-1α) signaling.^81^ HIF-1α subsequently drives macrophage polarization toward the pro-inflammatory M1 phenotype by modulating the Notch pathway and CCL2/C–C motif chemokine receptor 2 (CCR2) axis.^81^ Concurrently, the initial Ca^2+^ signal directly stimulates NF-κB signaling, thereby promoting M1 activation and secretion of inflammatory mediators.^82^^,^^83^ Piezo1 also engages the PI3K/Akt pathway, enhancing macrophage proliferation and further shaping the local inflammatory microenvironment.^84^ Collectively, mechanical stimulation-induced Piezo1 activation orchestrates immune cell polarization and modulates the periodontal immune microenvironment, thereby playing a pivotal role in regulating OTM.

### Function in neurons during OTM

Recent evidence has increasingly highlighted the influence of crosstalk between nerves and bones in the OTM process.^49^ Upon exposure to orthodontic forces, peripheral sensory nerves can facilitate an inflammatory cascade response and enhance osteoclast activity by expressing substance P (SP) and calcitonin gene-related peptide (CGRP).^85^ Furthermore, they facilitate the differentiation of PDLCs toward osteogenesis by expressing semaphorin 3A (Sema3A).^86^ Blocking these sensory nerves locally has been found to decelerate OTM.^85^ Local ablation of transient receptor potential vanilloid 1 (TRPV1)-expressing trigeminal nerves reduces osteoclast activation and slows the rate of tooth movement.^37^ However, genetic knockout of TRPV1 does not alter OTM, indicating that the TRPV1 channel itself is not directly involved in sensory nerve-mediated bone remodeling.^37^ In contrast, emerging evidence demonstrates that Piezo2 plays a predominant role in the crosstalk between sensory nerves and alveolar bone. Piezo2 is expressed in the majority of sensory nerves,^87^ and chemical silencing of Piezo2-expressing trigeminal afferent fibers markedly inhibits alveolar bone remodeling.^37^ Furthermore, conditional knockout of Piezo2 specifically in TRPV1-positive afferent nerves significantly slows tooth movement,^37^ suggesting that Piezo2 in both TRPV1-positive and TRPV1-negative trigeminal neurons contributes to OTM regulation. By transducing mechanical stimuli into biological signals, Piezo2 channels mediate nerve–bone communication and thereby influence OTM.

Taken together, the available evidence indicates that Piezo channels are involved in the mechanotransduction of OTM, while further research is needed to thoroughly establish its specific regulatory mechanisms.^88^ No abnormal tooth movement rate in the Piezo1^*Dmp1Cre*^mice OTM model was observed,^88^ but the local administration of Piezo1 inhibitors in the periodontium resulted in a noteworthy reduction in the tooth movement rate.^35^^,^^36^ These differences indicate the intricacy of OTM as a complex cellular process involving numerous interactions. In conclusion, Piezo channels modulate bone formation, bone resorption, and local inflammation by orchestrating the biological activities of diverse cell populations, playing a pivotal role in OTM.

### Association with orthodontic treatment complications

#### *Piezo is associated with root resorption*

Root resorption is deemed the most unfortunate complication of orthodontic treatment.^89^ Excessive orthodontic force, prolonged force duration, stress concentration due to root morphology, along with increased susceptibility to orthodontic forces resulting from factors such as age and genetics, are all considered risk factors for root resorption.^89^, ^90^, ^91^ Fundamentally, root resorption reflects an imbalance between mechanical loading and the reparative capacity. As the principal mechanosensory element in periodontal tissue, Piezo1 plays a crucial role in this process. Excessive orthodontic stress or disruption of the loading–unloading cycle can lead to overactivation of Piezo1 channels. This aberrant activation triggers inflammatory signaling cascades and drives the excessive release of osteoclastogenic factors.^65^ Such changes disrupt osteoclast–osteoblast coupling and alter the M1/M2 macrophage polarization ratio,^65^ culminating in local inflammatory overload. Collectively, these pathological events promote the onset and progression of root resorption. Age-related declines in Piezo1 expression have been documented in osteoblasts and osteocytes.^92^ In parallel, expression of *Tnfrsf11b*, the gene encoding the anti-osteoclastogenic protein OPG, is reduced both *in vitro* and *in vivo* under conditions of diminished Piezo1 levels in osteocytes.^92^ This mechanism may explain why the risk of root resorption increases with age. Moreover, Piezo channels are also expressed directly in cementoblasts and cementocytes.^88^ Unlike other cells mentioned above that inhabit periodontal tissue, murine cementoblasts show a decrease in Piezo1 expression when subjected to compressive force stimulation.^93^ Static compression can down-regulate cementogenic activity by inhibiting Piezo1.^93^ In summary, excessive orthodontic pressure, acting through Piezo1, simultaneously disrupts inflammatory homeostasis and impairs regenerative capacity. By driving both inflammatory overload and compromised tissue repair, Piezo1 emerges as a critical molecular target for the prevention and regulation of orthodontically induced root resorption.

### Piezo mediates orthodontic pain

Orthodontic pain is a common clinical challenge, affecting nearly all orthodontic patients.^94^^,^^95^ Similar to root resorption, excessive orthodontic pain primarily arises from a localized inflammatory response triggered by mechanical forces.^96^ Excessive mechanical loading activates Piezo1, which promotes the release of inflammatory mediators that sensitize peripheral nerve endings, thereby amplifying pain perception. For example, compressive stimulation of Piezo1 in PDLCs induces ATP release,^97^ which subsequently activates neuronal P2X3 receptors to elicit pain.^96^ Piezo2, a well-established mechanosensory channel involved in mechanical pain,^98^ has been linked to corneal pain, gastrointestinal hypersensitivity, and orofacial pain.^99^, ^100^, ^101^, ^102^ Experimental evidence shows that inferior alveolar nerve injury up-regulates Piezo2 expression in both the trigeminal ganglion and the subnucleus caudalis of the spinal trigeminal nucleus.^100^ Pharmacological blockade of Piezo2 markedly alleviates orofacial inflammatory pain.^100^ However, direct involvement of Piezo2 in orthodontic pain has not yet been confirmed, representing a critical avenue for future investigation. In conclusion, the mechanosensitive nature of Piezo channels underscores their potential as novel targets for orthodontic pain management.

### Prospects for application in orthodontic treatment

**Figure 3:**
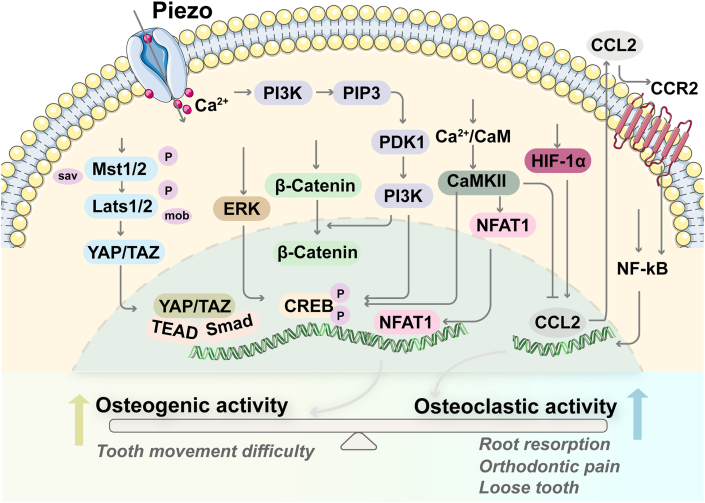
Piezo1 and downstream pathways regulate the balance between osteogenesis and osteoclastogenesis in periodontal tissue during orthodontic tooth movement. Orthodontic forces activate the Piezo1 channel, inducing Ca^2+^ influx. This activation triggers downstream Wnt/β-catenin, YAP/TAZ, ERK, PI3K/Akt, and CaMKII pathways, promoting the transcription of osteogenic factors. It also drives macrophage inflammatory polarization through the CCL2/CCR2 and NF-κB pathways, activating osteoclastogenesis. MST1/2, mammalian sterile 20-like kinase; LATS1/2, large tumor suppressor kinase 1/2; CaM, calmodulin; CaMKII, calmodulin-dependent protein kinase II; NFAT1, nuclear factor of activated T cells 1; HIF-1α, hypoxia-inducible factor-1α; CCL2, C–C motif chemokine 2; CCR2, C–C motif chemokine receptor 2; ERK, extracellular signal–regulated kinase; NF-κB, nuclear factor κB; YAP, yes-associated protein; TAZ, PDZ-binding motif; Akt, protein kinase B; PI3K, phosphatidylinositol-3 kinase.

Piezo converts mechanical stimuli into complex biochemical signals, regulating biological behaviors such as osteogenesis, osteoclastogenesis, and immune responses, thereby maintaining the balance between osteogenic and osteoclastic activity. Once the function of Piezo and its downstream pathways is dysfunctional or excessively activated, this balance will be disrupted, leading to a series of complications (Fig. 3). The significant involvement of Piezo channels in OTM and orthodontic complications provides a broad prospect for clinical applications.

### Piezo accelerates OTM

The therapeutic paradigm in contemporary orthodontics is undergoing continuous refinement alongside advances in technology. Adult patients, who increasingly constitute the majority of the treated population, tend to prioritize higher treatment efficiency and are more sensitive to the overall duration and cost of care. Consequently, clinical priorities have shifted from focusing exclusively on treatment outcomes toward a dual emphasis on efficacy and time efficiency.^103^ Within this context, accelerated tooth movement techniques have garnered growing clinical attention due to their potential to markedly reduce treatment duration and enhance patient satisfaction.^103^

Among these, corticotomy is widely recognized as one of the most effective acceleration methods,^104^ however, its invasive nature limits patient acceptance and elevates overall treatment costs. Alternative strategies, including local administration of prostaglandins and their analogs, as well as physical modalities such as pulsed mechanical vibration or photobiomodulation therapy, have demonstrated some capacity to facilitate tooth movement in experimental settings.^105^^,^^106^ Yet, both their efficacy and reproducibility remain controversial in clinical practice.^105^^,^^106^ For physical therapies, particularly the absence of standardized, biologically grounded parameter guidelines hampers consistency in treatment outcomes.

Piezo channels can be activated or sensitized by diverse physical stimuli, including mechanical vibration and thermal energy, thereby exerting direct regulatory effects on bone remodeling. Harnessing the biophysical properties of Piezo channels, such as their optimal activation thresholds and specific response frequency, may enable reverse engineering of parameter systems for physical therapy devices. This approach could increase the precision, predictability, and safety of clinical interventions. Compared with traditional pharmacological approaches that mainly promote osteoclastic activity, targeted modulation of Piezo channels offers the distinct advantage of bidirectional regulation. Piezo targeted therapy can coordinately enhance osteogenesis, thereby supporting balanced coupling between bone resorption and formation.

### Piezo functions in the treatment of orthodontic complications

Given the extensive involvement of Piezo channels in the pathogenesis of orthodontic complications, novel therapies targeting Piezo hold promise for providing precise treatment strategies. One promising avenue involves the development of peptide-based inhibitors with enhanced subtype selectivity and stability. Coupled with localized delivery systems, these agents could effectively suppress Piezo channel activation and subsequent downstream signaling, thereby slowing the progression of disease. In the context of pain management, Piezo-targeted therapeutics could establish a novel analgesic pathway distinct from conventional non-steroidal anti-inflammatory drugs. This dual approach may include locally administered Piezo1 inhibitors to attenuate the production of pro-nociceptive mediators within periodontal tissues. Piezo2-specific antagonists are engineered to penetrate the nerve sheath, directly blocking mechanically evoked pain signals in the peripheral nervous system.

In summary, a Piezo-centric therapeutic paradigm represents a breakthrough opportunity for managing dentofacial deformity, offering a mechanism-based framework for precision medicine in this clinical domain.

### Effects of Piezo channels in dentofacial deformity-associated diseases

#### *Affecting dentofacial deformity-associated TMJOA*

Patients with dentofacial deformities are at a heightened risk of developing temporomandibular joint disorders.^7^ Among these, TMJOA represents the most severe subtype, with malocclusion identified as a contributory risk factor in its development.^7^ The joint degeneration and pain associated with dentofacial deformities are primarily mechanical in nature, attributable to the mechanical overloading of the joint.^107^ Notably, Piezo channels that primarily respond to pathologic and destructive mechanical stimuli^14^ are key factors in mediating mechanical arthritis.

**Figure 4:**
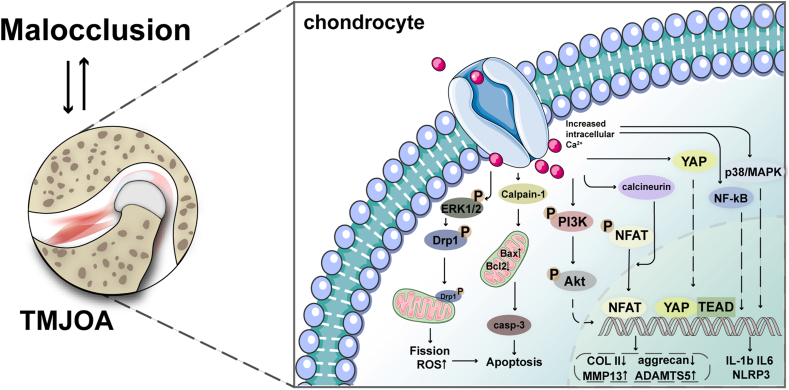
The role of Piezo1 and downstream pathways in chondrocytes of dentofacial deformity-associated temporomandibular joint osteoarthritis (TMJOA). When subjected to overload mechanical stimulation, chondrocyte Piezo1 is activated, leading to an excessive influx of Ca^2+^. This leads to mitochondrial dysfunction, Bcl-2/Bax/Caspase-3 signaling pathway activation, and subsequent apoptosis. Elevated intracellular calcium levels also trigger the activation of the PI3K/Akt signaling pathway or induce the translocation of NFAT and YAP into the nucleus, ultimately causing the loss of cartilage characteristics in chondrocytes. Moreover, it promotes the activation of the p38/MAPK and NF-κB pathways, releasing inflammatory factors. NFAT, nuclear factor of activated T cells; ERK, extracellular signal–regulated kinase; NF-κB, nuclear factor κB; YAP, yes-associated protein; Akt, protein kinase B; PI3K, phosphatidylinositol-3 kinase; ROS, reactive oxygen species; Drp1, dynamin-related protein 1; Bax, Bcl2-associated X protein; Bcl2, B-cell lymphoma-2; Casp-3, cysteine-dependent aspartate-specific protease-3.

Studies have demonstrated a significantly increased expression of Piezo1 in the condylar cartilage of the rat model of unilateral anterior crossbite, simulating malocclusion-induced TMJOA^108^ (Fig. 4). In this abnormal mechanical environment, the overexpression of Piezo1 leads to intracellular calcium overload,^109^ subsequently triggering mitochondrial calcium uptake and oxidative phosphorylation dysfunction.^11^^,^^109^^,^^110^ As a result, chondrocytes undergo senescence and even apoptosis.^11^^,^^109^^,^^110^ Additionally, increased intracellular calcium influx regulates chondrocyte activity by activating downstream channels. For example, it triggers the PI3K–Akt pathway and facilitates NFAT and YAP nuclear translocation, driving chondrocytes toward terminal differentiation.^111^, ^112^, ^113^ This is evidenced by the reduced expression of SRY-box transcription factor 9 (Sox9) and collagen type II alpha 1 chain (Col2a1) and an elevation in matrix metallopeptidase 13 (Mmp13) expression, leading to cartilage matrix degradation.^111^, ^112^, ^113^ Furthermore, activating p38 mitogen-activated protein kinase (p38/MAPK) and NF-κB pathways releases inflammatory factors, generating an inflammatory environment within the cartilage.^111^^,^^114^^,^^115^ This localized inflammation can subsequently increase the mechanosensitivity of chondrocytes by up-regulating Piezo1 expression,^116^ creating a vicious cycle of degeneration. Based on the evidence, Piezo1 is deemed a promising target for arthritis therapy, as demonstrated in preclinical animal models.^108^^,^^117^^,^^118^ Drugs such as urocortin 1 (Ucn1) and artemisinin have been found to exert therapeutic effects by inhibiting Piezo1 channels.^113^^,^^119^

While a study has demonstrated that maladaptive chondrocyte responses to excessive mechanical stimuli depend on the synergy between Piezo1 and Piezo2, both Piezo1 and Piezo2 are expressed in chondrocytes.^110^ However, other studies have found a predominance of Piezo1 expression in chondrocytes rather than Piezo2.^14^ Due to this discrepancy in expression within cartilage, current research on Piezo channels has primarily focused on examining the role of Piezo1 in articular cartilage degradation. On the other hand, Piezo2, expressed in nociceptors, has been implicated in arthritis pain.^120^ Nociceptor-specific knockout mice for Piezo2 have effectively relieved pain and mechanical sensitivity associated with arthritis.^120^ However, these studies have primarily focused on the knee and dorsal root ganglia.^120^ Further research is necessary to establish whether Piezo2 plays a comparable role in the trigeminal ganglion, which innervates TMJOA pain.

### Associated with dentofacial deformity-associated periodontitis

Periodontitis is an infectious disease initiated by bacterial plaque.^121^ Dentofacial deformities, including malocclusion, particularly crowding, complicate oral cleaning, accumulating plaque,^122^ which consequently elevates the risk of periodontitis. Furthermore, malocclusion, when accompanied by occlusal trauma, can exacerbate the progression of periodontal destruction in the context of pre-existing inflammation.^6^^,^^123^ Studies have shown a significant increase in the RANKL/OPG ratio in MC3T3-E1 cells co-treated with *Porphyromonas gingivalis* lipopolysaccharides and mechanical stress compared with those treated separately.^124^ This confirms that individuals with malocclusion accompanied by plaque accumulation and abnormal mechanical stimulation face an elevated risk of periodontal bone destruction. In fact, periodontitis represents a pathological process involving multiple cell types. Bacterial accumulation up-regulates local Piezo expression,^125^ thereby inducing a periodontal mechanosensitive state. Excessive Piezo activation disrupts the balance between osteoclasts and osteoblasts, promotes inflammatory polarization of immune cells, and ultimately leads to alveolar bone destruction.^126^ Notably, the osteoclast effects caused by *Porphyromonas gingivalis* lipopolysaccharides or mechanical stress can be mitigated by Piezo1 knockdown.^124^

Therefore, based on the mechanistic pathologies associated with dentofacial deformity-related diseases, the Piezo channel is a reliable target for their treatment. The development of novel drugs targeting the Piezo channel also shows great promise in the treatment of related diseases.

## Conclusion and perspective

Recent studies have shed further light on the structure and function of Piezo channels. Piezo channels are being extensively researched as a potential catalyst for dentofacial deformity. By mapping the roles of Piezo channels across the entire “pathology–treatment–complication” continuum in dentofacial deformities, this review highlights their multifaceted functions as central mechanosensors. These channels orchestrate bone remodeling, local immunity, and peripheral neurocrosstalk within the mechanical microenvironment. This understanding is crucial not only for deciphering disease mechanisms but also for informing the development of next-generation dentofacial therapies capable of concurrently promoting tissue remodeling, suppressing inflammation, and alleviating pain.

## CRediT authorship contribution statement

**Jinhan Nie:** Writing – original draft, Investigation, Data curation, Conceptualization. **Yi Zhang:** Supervision, Project administration, Investigation. **Jun Ma:** Resources, Project administration, Formal analysis. **Min Hu:** Writing – review & editing, Supervision, Investigation. **Huichuan Qi:** Writing – review & editing, Supervision, Investigation, Funding acquisition.

## Data availability

The data presented in this study are available on request from the corresponding author.

## Fundings

This study was supported by the 10.13039/501100001809National Natural Science Foundation of China (No. 82100956), the 10.13039/100007847Jilin Provincial Natural Science Foundation of China (No. YDZJ202301ZYTS432), and the IOF Young Research Grant for HQ (China) (No. IOF2023Y19).

## Conflict of interests

The authors declared no conflict of interests. The funders had no role in the design of the study, in the collection, analyses, or interpretation of data, in the writing of the manuscript, or in the decision to publish the results.
